# Flood sensitivity assessment of super cities

**DOI:** 10.1038/s41598-023-32149-8

**Published:** 2023-04-05

**Authors:** Zijun Wang, Xiangyu Chen, Zhanshuo Qi, Chenfeng Cui

**Affiliations:** 1 College of Water Resources and Architecture Engineering, Northwest A&F University, Yangling, Xianyang, 712100 China; 2 Key Laboratory of Agricultural Soil and Water Engineering in Arid and Semiarid Areas of Ministry of Education, Northwest A&F University, Yangling, Xianyang, 712100 China

**Keywords:** Hydrology, Natural hazards

## Abstract

In the context of global urbanization, more and more people are attracted to these cities with superior geographical conditions and strategic positions, resulting in the emergence of world super cities. However, with the increasing of urban development, the underlying surface of the city has changed, the soil originally covered with vegetation has been substituted by hardened pavement such as asphalt and cement roads. Therefore, the infiltration capacity of urban rainwater is greatly limited, and waterlogging is becoming more and more serious. In addition, the suburbs of the main urban areas of super cities are usually villages and mountains, and frequent flash floods seriously threaten the life and property safety of people in there. Flood sensitivity assessment is an effective method to predict and mitigate flood disasters. Accordingly, this study aimed at identifying the areas vulnerable to flood by using Geographic Information System (GIS) and Remote Sensing (RS) and apply Logistic Regression (LR) model to create a flood sensitivity map of Beijing. 260 flood points in history and 12 predictors [elevation, slope, aspect, distance to rivers, Topographic Wetness Index (TWI), Stream Power Index (SPI), Sediment Transport Index (STI), curvature, plan curvature, Land Use/Land Cover (LULC), soil, and rainfall] were used in this study. Even more noteworthy is that most of the previous studies discussed flash flood and waterlogging separately. However, flash flood points and waterlogging points were included together in this study. We evaluated the sensitivity of flash flood and waterlogging as a whole and obtained different results from previous studies. In addition, most of the previous studies focused on a certain river basin or small towns as the study area. Beijing is the world's ninth largest super cities, which was unusual in previous studies and has important reference significance for the flood sensitivity analysis of other super cities. The flood inventory data were randomly subdivided into training (70%) and test (30%) sets for model construction and testing using the Area Under Curve (AUC), respectively. The results turn out that: (1) elevation, slope, rainfall, LULC, soil and TWI were highly important among these elements, and were the most influential variables in the assessment of flood sensitivity. (2) The AUC of the test dataset revealed a prediction rate of 81.0%. The AUC was greater than 0.8, indicating that the model assessment accuracy was high. (3) The proportion of high risk and extremely high risk areas was 27.44%, including 69.26% of the flood events in this study, indicating that the flood distribution in these areas was relatively dense and the susceptibility was high. Super cities have a high population density, and once flood disasters occur, the losses brought by them are immeasurable. Thus, flood sensitivity map can provide meaningful information for policy makers to enact appropriate policies to reduce future damage.

## Introduction

The construction of super cities in the twenty-first century have become a weathervane of urban development all over the world. However, it is followed by a higher risk of natural disasters, such as the flood disasters in this study. In super cities with greater urban population density and higher ground hardening rate, the implementation of flood sensitivity analysis is of great significance^1^. Urban waterlogging occurs in a short time when continuous rainfall or heavy rainfall exceeds the excretion capacity of the city, which will lead to the formation of urban waterlogging. It is a natural disaster often suffered by the main urban areas of super cities^2^. Flash flood often happens in mountainous areas. It is characterized by sudden, concentrated water volume, high velocity, strong erosion damage, and sediment or even rocks carried in the water flow^3^, which is a common natural disaster in the suburbs of super cities^4,5^.

Here in the world, many cities are built on plains or basins surrounded by mountains. The center of the plain or basin is usually the center of the city. With the construction of urbanization, it continues to radiate outward^6–9^. Suburbs around the main urban areas are usually built around mountains, clustered at river passes. The main metropolitan area of the city is severely affected by waterlogging^1^, while the surrounding suburbs are also under constant threat of flash flood^10^. In addition, global warming leads to more frequent extreme rainfall events and more flooding events, leading to various dangerous phenomena associated with the corresponding problems^11^. In the first half of 2022 alone, flooding affected 21.805 million people in China, resulting in direct financial losses of 64.76 billion yuan. In July 2021, the city cluster centered on Zhengzhou in Henan province has been hit by heavy rain, and daily precipitation is as high as 552.5 ml, leaving 398 people dead or missing^12,13^; Each year during the rainy season, flash floods in southern and western China cause a large number of casualties. The “2022 Aug. 13” flash flood in Pengzhou, Sichuan Province caused 7 deaths, and the “2022 Aug. 18” flash flood in Datong County, Qinghai Province has caused 16 deaths so far. Therefore, the current research urgently needs to carry out simulation research in the regional scale space, so as to reduce or even prevent the negative impact of floods^14–16^. Flood can be divided into river floods, coastal floods, waterlogging and flash floods and other types (hereinafter collectively referred to as floods) depending on their mechanism^17^. In the construction of super cities, flood control measures should be formulated in advance according to the flood sensitivity map, considering the great flood damage^18^. Therefore, the assessment of flood sensitivity is expected to receive further continuous attention in the future.

Flood sensitivity refers to the possibility of flooding in an area under conditions such as local topography. Flood sensitivity maps can be used to predict where flooding is likely to occur. Flood sensitivity assessment is the important precondition for the flood prevention and control work. For the past few years, the rapid development of GIS has combined GIS-based numerical simulation with statistical methods, resulting in the use of weighted indices to draw flood sensitivity maps. Among them, the common methods include Analytic Hierarchy Process (AHP)^19–22^, Frequency Ratio (FR)^23,24^, Weight Of Evidence (WOE)^24,25^, Logistic Regression (LR)^23^, weighting factor, etc. However, with the further development of machine learning, some newer methods have emerged and been applied to spatial modeling of flood sensitivity, and gratifying results have been obtained. At first, it was the application of a single algorithm. For instance, Artificial Neural Network (ANN)^26–28^, Support Vector Machine (SVM)^29^, Naive Bayesian^30^, Random Forest^25^, Decision Tree^31^ and neuro-fuzzy methods^31^, etc. Recently, in order to further improve the prediction accuracy of the model, a variety of mixed model methods have been introduced^32–34^, for example Genetic Algorithm (GA)^35^, Particle Swarm Optimization (PSO)^36^, Biogeographic-based Optimization (BBO), Bat Algorithm (BA)^37^, Ant Colony Optimization (ACO)^38^, Firefly Algorithm (FA)^39^, etc. Similarly, ANN has been combined with FA and Levenberg–Marquardt backpropagation algorithms to generate flood sensitivity maps^10^. According to previous studies, deep learning algorithm models are superior to traditional models in multiple fields of study^40–42^.

While advanced machine learning algorithms have been used to assess flood sensitivity, the classical LR model was still used. The reason is that LR has been proven to be effective for disaster sensitivity mapping. Moreover, LR has shown many advantages in data processing and result representation. For example, the independent variables in LR need not be normally distributed, and the result output of LR can be very effective in detecting the accuracy of sample data. Therefore, we believe that LR can meet the performance requirements of this study, such as prediction accuracy, ranking of impact factors, and probability estimation. In this paper, the super cities of Beijing is taken as an example. Under the background of urbanization, the flood sensitivity assessment is carried out based on GIS and LR, the importance of predictive variables is calculated, the model performance is verified by ROC curve, and flood sensitivity map is drawn, supplemented by flood observation records. Different from previous studies, most of them separate flash flood from waterlogging separately^43–45^, but in fact, cities include both the central main urban area and the surrounding mountains and suburbs, especially the super cities with rapid development in recent years. Therefore, the novelty of the study is that the map of flood sensitivity covers flash floods and waterlogging, and the sensitivity assessment of flash flood and waterlogging as a whole is carried out to explore the flood sensitivity under the joint effect of the two factors, because they are both important disaster contents of the super cities.

## The study area and flood inventory map

**Figure 1:**
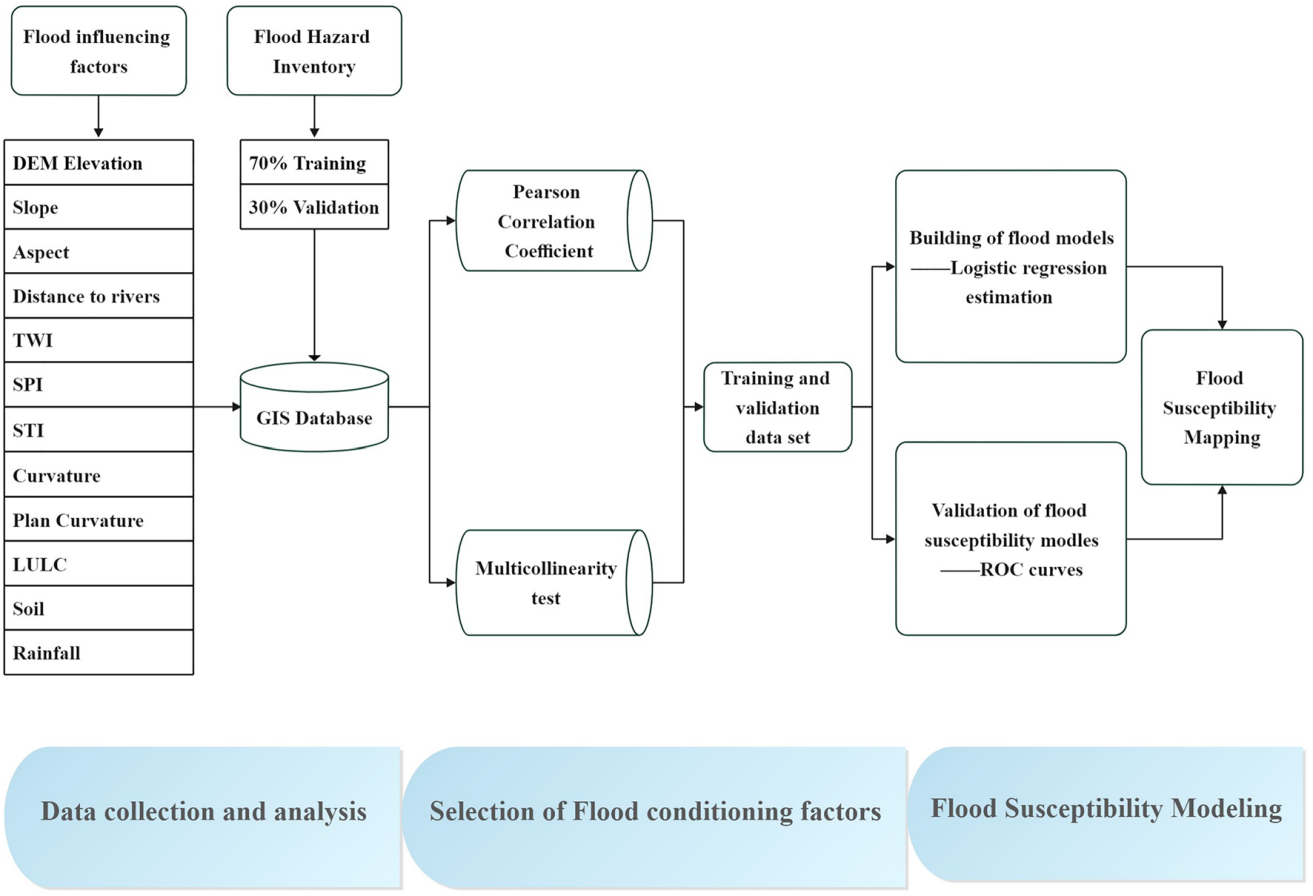
The working framework for flood sensitivity analysis.

The whole work is summarized by defining and drawing the working framework in the process of flood sensitivity analysis, including flood sensitivity map, flood predictor generation, flood sensitivity model modeling using Logistic Regression, model evaluation, etc. (Fig. 1).

### The study area

Beijing, the capital of China, is located at 115.7°–117.4° E and 39.4°–41.6° N. The total area is 16,410 km^2^, of which the mountain area is 10,200 km^2^, accounting for 62% of the total, and the rest is plain. Urbanization has made the urban built-up area of Beijing grow from 109 square kilometers in 1949 to 1289.3 km^2^ in 2022, and it is still showing a trend of further expansion. The population of permanent residents of the city has reached 21.89 million, becoming China's second largest city, the ninth in the world's most populous city. The northwest of Beijing is mostly mountainous, and the southeast is mostly plain. Beijing is located in the northern part of the North China Plain, adjacent to the Bohai Bay, bounded by the Taihang Mountains to the west and Yanshan Mountains to the north and northeast, with an average elevation of 43.5 m. The vegetation type is mostly deciduous broad-leaved forest. The rock types in Beijing can be divided into loose sedimentary rocks and hard rocks (bedrock). The loose deposits are mainly distributed in the piedmont plain, and hard rocks are mainly exposed in the mountainous area, including magmatic rocks, metamorphic rocks and sedimentary rocks. The precipitation in Beijing is abundant and the seasonal distribution is not uniform. June, July, August concentrated 80% of the annual precipitation, it's easy to have a small high strength heavy rain in July and August. The whole area of Beijing was selected as the research area of this paper. The built-up areas of Beijing have the most frequent waterlogging, especially the low-lying areas, sunken overpasses, underground passageways, dilapidated buildings and construction sites. The surrounding suburban villages are close to mountains and water systems, especially in flood season, where flash flood seriously affected the lives and property safety of villagers. As a representative of the world's super cities, the analysis of flood sensitivity of Beijing is also of reference significance to the flood control planning of other super cities.

### Flood inventory map

**Figure 2:**
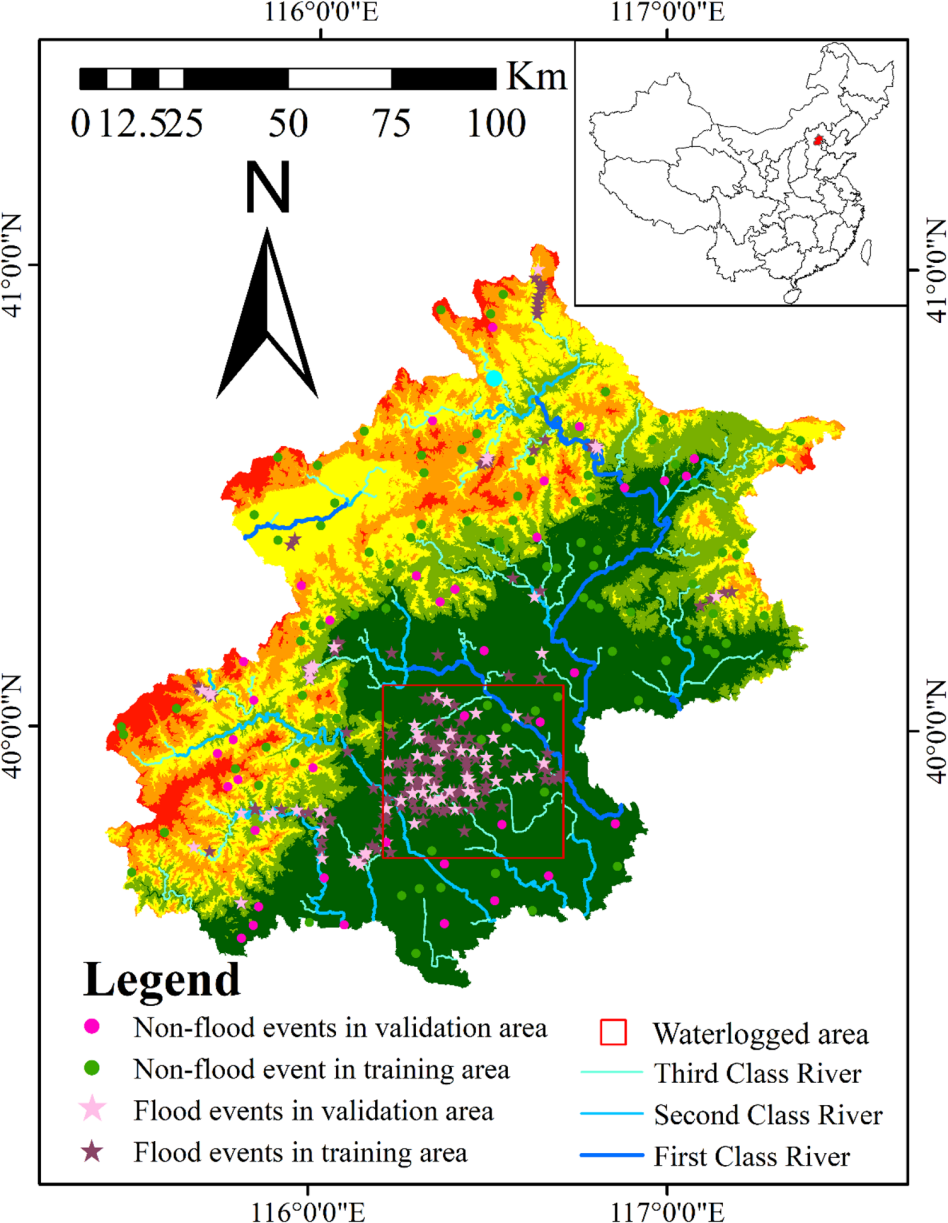
Map of flood and non-flood locations (we plotted the map using arcgis10.7. http://www.esri.com/sofware/arcgis).

Flood inventory maps are a critical first step in sensitivity assessment. In this paper, we studied the flood events in the history of Beijing from 2012 to 2022 with severe disasters, especially focusing on the rainstorm event in Beijing on July 21, 2012, which caused 79 deaths, collapsed 10,660 houses, 1.602 million people were affected, and 11.64 billion yuan financial losses was caused. This is the worst rainstorm and flood disaster in Beijing and surrounding areas in 61 years^46^. The flood inventory map is based on information posted by various social media, local governments and municipal authorities, with a total of 260 flood disaster spots. Non-flood sites should also be considered when generating datasets, as flood sensitivity assessment is a binary classification problem^47^. For purpose of ensuring the accuracy, 130 non-flood points were generated based on ARCGIS 10 randomly. 70% project randomly selected for training area, 30% of the projects selected randomly to test area. The flood points in the flood inventory were geolocated in the digital map using ARCGIS 10.8, and the value was assigned as 1, indicating the presence of flood, no flooding points were set to 0. The scope of the main urban area of Beijing was identified from the Google satellite ground, and judged according to the text description and picture report of the flood disaster by social media, local government and municipal authorities. The areas with frequent waterlogging were basically marked with red lines (Fig. 2).

## Methodology

### Data sources

Land use remote sensing monitoring data, soil texture spatial distribution data of China, precipitation spatial interpolation data, and nighttime light data are all from the free data released on the Internet by the Resource and Environmental Science and Data Center, Institute of Geographic Sciences and Natural Resources Research, Chinese Academy of Sciences. The geographic elevation data were gained from ASTER GDEM database of Geospatial Data Cloud platform with a resolution of 30 m. The other predictors were obtained by using "raster calculator" in ARCGIS 10.8 toolbox on the basis of DEM data in the study area. Flood inventory were obtained from various social media, local governments and municipal authorities (Table 1).

**Table 1:** Table of data use.

Data	Format	Resolution	Time	Source
Flood points	Shape file	Point data	2012–2022	Network news and publicity
Land use	Raster file	1000 m	2020	https://www.resdc.cn/Default.aspx
Soil	Raster file	1000 m	Background data	https://www.resdc.cn/Default.aspx
Rainfall	Raster file	1000 m	2015	https://www.resdc.cn/Default.aspx
DMSP-OLS night light data	Raster file		2013	https://www.resdc.cn/Default.aspx
DEM	Raster file	30 m	2022	ASTER GDEM (http://www.gscloud.cn)

### Selection of flood predictors

**Figure 3:**
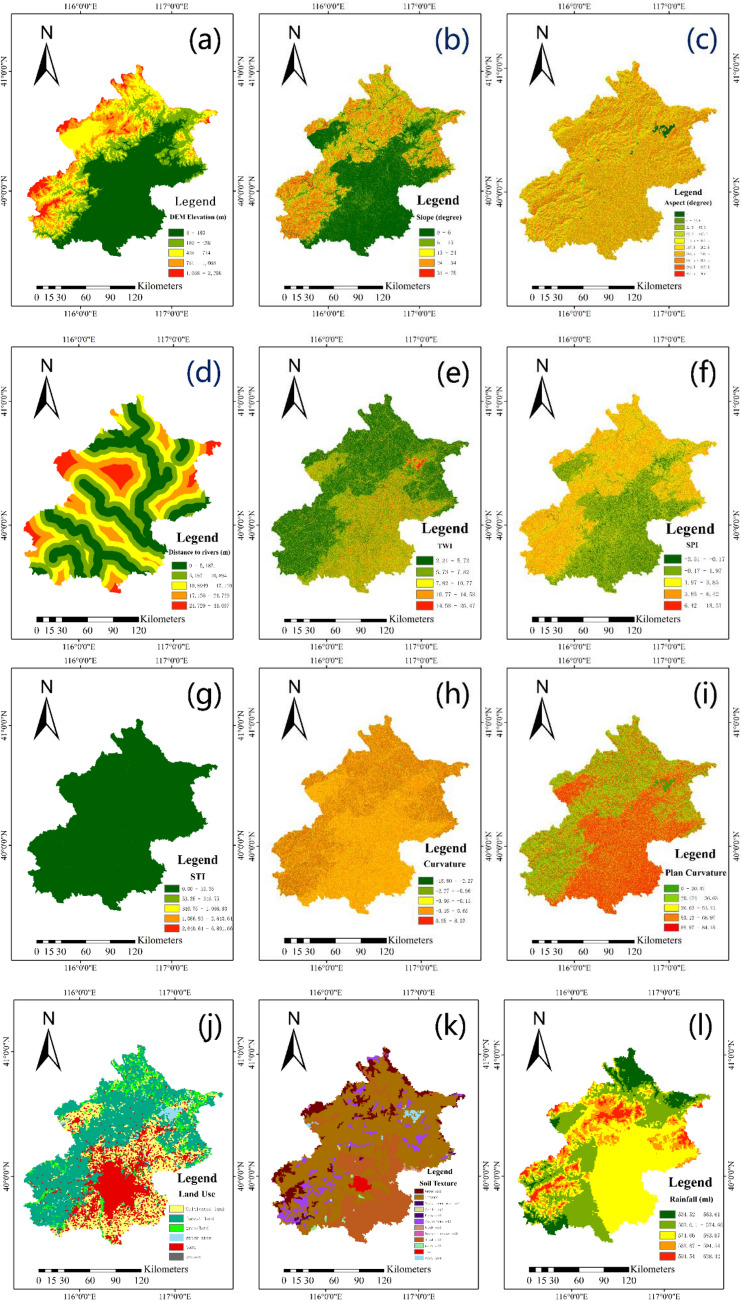
Predictors: (**a**) Dem elevation, (**b**) slope, (**c**) aspect, (**d**) distance to rivers, (**e**) TWI, (**f**) SPI, (**g**) STI, (**h**) curvature, (**i**) plan curvature, (**j**) LULC, (**k**) soil, (**l**) rainfall (we plotted the map using arcgis10.7. http://www.esri.com/sofware/arcgis).

Finding predictors for flood model construction is important and complex. Scientific and reasonable selection of parameters is conducive to improving the accuracy of flood sensitivity map. Combined with previous literature, in this study, 12 flood predictors were used: DEM, slope, aspect, distance to rivers, TWI, SPI, STI, curvature, plan curvature, LULC, soil, and rainfall^45,48^. The "resampling" toolbox in ARCGIS 10.8 was used to transform all the influencing factors into a 30 m spatial resolution raster format (Fig. 3).

#### DEM elevation

Elevation is a key factor in whether or not a flood occurs^49–51^. Physical meaning of using elevation is, the water is always under the influence of gravity shift from high to low altitude, so lower altitude increases the chances of flooding, and vice versa^52,53^.

#### Slope

The speed at which the flood moves depends on the slope^54^. Large slope will cause the decrease of soil water holding capacity and infiltration capacity, thus accelerating the velocity of runoff, and vice versa. As a result, areas with low and flat terrain will have more opportunities for flooding and greater flood sensitivity^55^.

#### Aspect

Slope aspect influences the direction of flood movement while being able to maintain soil moisture, which indirectly affects the likelihood of flooding^56^.

#### Distance to rivers

Distance to rivers is an important factor affecting flood sensitivity^57^, on account of distance controls flood events and the flow of rivers into rivers^58^. In a rainstorm event, when the river level rises, the surrounding area is affected first, and the flood that overflows the embankment flows to different areas according to different slope directions. Therefore, the closer the distance to rivers, the more affected it is first, and the greater the flood sensitivity^59^.

#### TWI

TWI is an indicator of the impact of topography on runoff flow direction and accumulation, which can effectively spatially express the differences in watershed moisture^60,61^. TWI is the function of slope and the upstream contribution area, it helps to identify potential increase of regional rainfall runoff model, soil water content and water areas, according to the area every pixel size of the water^62^. This is calculated using Eq. (1):

1 $$\text{TWI}=\ln \left(\frac{\text{SCA}}{{\text{Slope}}} \right)$$

where SCA is the local upslope catchment that flows through the mesh cells, and Slope is the steepest outward slope per mesh cell, measured by drop/distance, the "tan" value of the slope angle.

#### SPI

SPI is usually used to describe the flow and erosion of a point on the surface. The increase of watershed area and slope will lead to the increase of water volume and flow velocity contributed by the upslope area, and the corresponding increase of SPI, thus increasing the risk of soil erosion. This is calculated using Eq. (2):

2 $${\text{SPI}} \, =\, \ln({\text{SCA*Slope}})$$

where SCA is the local upslope catchment that flows through the mesh cells, and Slope is the steepest outward slope per mesh cell, measured by drop/distance, the "tan" value of the slope angle.

#### STI

STI is a useful comprehensive topographic variable that characterizes the movement of water and sediment in a specific location. STI quantifies the location of a point in the landscape, which can increase the frequency of flooding and lead to the damage of the foundation^63^. The bed of a channel changes as sediment is deposited, reducing the capacity of the channel to hold water and leading to flooding. This is calculated using Eq. (3):

3 $$\text{STI} = {\left(\frac{\text{SCA}}{22.13} \right)}^{0.6}{\left(\frac{{\sin{\text{(S}{lope)}}}}{0.0896}\right)}^{1.3}$$

where SCA is the local upslope catchment that flows through the mesh cells, and Slope is the steepest outward slope per mesh cell, measured by drop/distance, the "tan" value of the slope angle.

#### Curvature

The magnitude of the curvature can be obtained by returning its radius value. Negative curvature values indicate concavity, positive curvature values indicate convexity, and zero curvature values indicate plane^64,65^. Curvature can influence the flood of water balance^37,66^, areas with negative values are prone to flooding, which is closely related to the convergence of runoff process^67^.

#### Plan curvature

Plane curvature refers to the curvature of the curve at that point on the terrain surface, specific to any point, through the point horizontal plane cutting the terrain surface in the horizontal direction. The plane curvature describes the bending and change of the surface along the horizontal direction, which is the bending degree of the ground contour line at the point. It is the slope analysis again based on the result of slope aspect analysis of DEM data in the study area.

#### LULC

The surface runoff and sediment transport were affected by LULC by controlling surface runoff generation and infiltration, thus LULC directly affected flood frequency^68^. Remote sensing data of land use in 2020 were used in this study, which was generated by manual visual interpretation based on Landsat TM images of the United States. We also divided LULC into six categories: cultivated land, forest land, grassland, water, town, and unused land. Since LULC did not change much in the short term, LULC data of 2020 was considered to be available.

#### Soil

Soil properties directly affect rainfall runoff infiltration, and the higher the permeability, the lower the chance of flooding^69^. The physical properties of the soil determine its water-holding capacity and also determine its ability to receive and drain water during rainfall events, indirectly affecting flood duration. The spatial distribution data of soil properties used in this study are 1:1 million soil type map and soil profile data obtained from the second soil census, which are background data.

#### Rainfall

Rainfall is an important condition leading to the occurrence of floods^70^. In this study, a spatially interpolated precipitation dataset in 2015 was used, which was located in the range of the time series studied. It was generated by collation, calculation and spatial interpolation processing based on daily observations from multiple meteorological stations. Rainfall was interpolated using ANUSPLIN interpolation software from Australia. ANUSPLIN is a tool for analyzing and interpolating multivariate data by smoothing spline function, that is, a method of approximating surface by function, which can make reasonable statistical analysis and data diagnosis. The spatial distribution of data can also be analyzed and it can implement the function of spatial interpolation.

### Flood sensitivity modeling

In the probability analysis of predicting floods, Logistic Regression model is commonly used to perform a calculus of the probability of a disaster when the dependent variable is known^71^. In LR, considering that the conditional variable plays a leading role in determining the dependent variable, the effect of model fitting in the analysis becomes better as the number of independent conditional variables increases^72^.

LR has been shown to be effective in disaster sensitivity mapping in previous studies and has demonstrated many advantages in data processing and result representation^73–75^. For example, the independent variables in LR need not be normally distributed; The data type of the condition factor is not restricted; The result output of LR can be very effective in detecting the accuracy of the sample data^76^. Therefore, it is considered that LR can meet our requirements for prediction accuracy, ranking of impact factors and performance of probability estimation^77^.

Used for flood sensitivity analysis in this study, LR is designed to describe the dependent variable and independent variable of the relationship between the best fitting model^24^. The occurrence of flood was used as the dependent variable to indicate the presence (value 1) or absence (value 0) of flood, which was used together with GIS to predict the likelihood of future floods^78^. Therefore, the Eq. (4) can be described as the correlation between flood occurrence and its dependence^79^:

4 $$\text{P} = \frac{1}{\text{1} + {\text{e}}^{-\text{z}}}{=}\frac{{\text{e}}^{\text{z}}}{\text{1}} + {\text{e}}^{\text{z}}$$

where, P is the probability of a flood. On the sigmoid curve line, the probabilities vary from 0 to 1, and Z is a linear combination. Thus, LR involves fitting Eq. (5) of the form:

5 $$\text{Z} = \upbeta_{0} + \upbeta_{0} {\text{x}}_{\text{z}} + \upbeta_{1} {\text{x}}_{2} + \cdots + \upbeta_{{\text{n}}} {\text{x}}_{{\text{n}}}$$

where Z is the combined effect of the flood, X_i_ (i = 1,2,……,n) are flood impact factors, β_0_ is the model intercept, and β_i_ is the parameter of the LR model^80^.

## Results and discussions

### Pearson correlation coefficient

In natural science, Pearson correlation coefficient is widely used to measure value between 1 to 1 between the two variables. This is calculated using Eq. (6):

6 $$\text{R} = \frac{\sum_{\text{i=1}}^{\text{n}}{\text{(X}}_{\text{i}}-\stackrel{\mathrm{-}}{\text{X}}\text{)(}{\text{X}}_{\text{i}}-\stackrel{\mathrm{-}}{\text{X}}\text{)}}{\sqrt{\sum_{\text{i=1}}^{\text{n}}{\text{(}{\text{X}}_{\text{i}}-\stackrel{\mathrm{-}}{\text{X}}\text{)}}^{2}}\sqrt{\sum_{\text{i=1}}^{\text{n}}{\text{(}{\text{Y}}_{\text{i}}-\stackrel{\mathrm{-}}{\text{Y}}\text{)}}^{2}}}$$

where R represents the Pearson correlation coefficient between variables x and y, n is the number of variables x and y. Pearson correlation coefficient values corresponding to a specific level see Table 2^81^.

**Table 2:** The Pearson correlation coefficient value (R) and corresponding correlation levels.

R	Correlation levels
|R|= 0	Absence of correlation
0 <|R|< 0.2	Very weak correlation
0.2 <|R|< 0.4	Weak correlation
0.4 <|R|< 0.6	Medium correlation
0.6 <|R|< 0.8	Strong correlation
0.8 <|R|< 1	Very strong correlation
|R|= 1	Complete correlation

In the flood sensitivity analysis, we generated a correlation matrix (Table 3) by Pearson correlation test to measure the correlation between independent variables (flood predictors). We expect the independent variables to be perfectly correlated with themselves, which means that the correlations between the variables are poor. The results of the test show that DEM and slope, LULC; Plan curvature and slope; slope and SPI; SPI and STI have moderate linear correlations, which may lead to the linear relationship between the other factors was weak.

**Table 3:** Pearson's correlation matrix.

	DEM elevation	Distance to rivers	Plan curvature	Slope	Aspect	Curvature	Rainfall	SPI	STI	LULC	SOIL	TWI
DEM elevation	1											
Distance to rivers	0.150**	1										
Plan curvature	− 0.351**	0.009	1									
Slope	0.633**	0.109*	− 0.600**	1								
Aspect	0.012	0.055	0.030	0.054	1							
Curvature	0.020	0.028	− 0.333**	0.397**	0.055	1						
Rainfall	0.009	0.142**	0.106*	− 0.119*	0.055	0.043	1					
SPI	0.471**	0.001	− 0.343**	0.540**	0.076	0.155**	0.063	1				
STI	0.216**	− 0.136**	0.023	0.112*	0.051	0.081	0.004	0.576**	1			
LULC	− 0.554**	0.032	0.264**	− 0.475**	0.021	0.028	0.102*	− 0.339**	−0. 153**	1		
Soil	−0. 377**	0.026	0.238**	− 0.360**	0.025	0.093	0.071	− 0.253**	0.090	0.362**	1	
TWI	− 0.332**	− 0.168**	0.319**	− 0.569**	0.047	− 0.254**	0.071	0.048	0.302**	0.275**	0.279**	1

**Correlation is significant at the 0.01 level (2-tailed).

*Correlation is significant at the 0.05 level (2-tailed).

### Multicollinearity test

Multicollinearity means that there is a linear correlation between the independent variables of the regression model^82^. If there are multiple collinearity, when calculating the independent variables the partial regression coefficient of β, matrix is irreversible, has led to the infinite solution or no solution of β^83^. The multicollinearity test is performed to rule out this hidden risk. In a test, if found in an LR model, two or more independent variables highly correlated, which means that a variable can be from other variables linear prediction. It is worth mentioning that even if multicollinearity occurs, it does not reduce the reliability and predictive the powerful features of the model; it only affects the estimates associated with a single predictor variable.

There are many ways to test for multicollinearity, such as tolerance (TOL), variance inflation factor (VIF, reciprocal of TOL), pairwise scatter plots and eigenvalues in correlation matrices^84^. In the study, we use TOL and VIF to detect the multicollinearity (Table 4). VIF tests for multicollinearity by comparing the correlation of other explanatory variables with a given explanatory variable. VIF consists of a index, said the index calculation due to the multicollinearity and estimate the variance of the regression coefficient of how much more. The variance inflation factor can be calculated using Eqs. (7) and (8):

**Table 4:** Multicollinearity analysis of predictors.

	Unstandardized coefficients		Standardized coefficients	t	Sig.	Collinearity statistics	
	B	Std. error	Beta			Tolerance	VIF
(Constant)	7.259	1.284		5.655	0.000		
DEM elevation	0.000	0.000	0.318	4.821	0.000	0.433	2.308
Distance to rivers	7.116 e−05	0.000	0.034	0.748	0.455	0.889	1.125
Plan curvature	1.683 e−05	0.001	0.001	0.015	0.988	0.616	1.624
Slope	0.010	0.003	0.254	2.915	0.004	0.248	4.036
Aspect	0.000	0.000	0.044	1.000	0.318	0.963	1.038
Curvature	0.036	0.029	0.062	1.216	0.225	0.720	1.390
Rainfall	0.001	0.000	0.246	5.486	0.000	0.940	1.064
SPI	0.015	0.013	0.074	1.108	0.268	0.419	2.387
STI	0.004	0.007	0.032	0.544	0.587	0.534	1.874
LULC	0.009	0.002	0.304	5.537	0.000	0.626	1.599
Soil	0.001	0.000	0.137	2.804	0.005	0.786	1.273
TWI	0.002	0.010	0.015	0.251	0.802	0.511	1.956

7 $$\text{VIF} = \frac{1}{\text{1} - {{\text{R}}_{\text{i}}}^{2}}$$

8 $$\text{TOL} = \frac{1}{{\text{VIF}}}$$

Among them, the R is the negative correlation coefficient between the independent variables and the other independent variables in the regression analysis.

The possibility of collinearity among independent variables increases with the increase of VIF. Based on previous experience, when the VIF exceeds 10, it indicates that the regression model has severe multicollinearity. When the TOL is greater than 0.1, the range of VIF less than 10 is acceptable, shows that there exists no problem of collinearity among the independent variables. If the VIF is greater than 10, then the general linear model is not applicable and is usually remedied by dropping variables with large VIF or combining related variables into a single variable. Table 4 shows that the TOL values of all independent variables in this study were greater than 0.1 and the VIF values were less than 10, which means that the VIF values of all independent variables did not have the problem of multicollinearity. Therefore, all of these variables were included in the LR and tested, as each variable could have an impact on flooding.

### Logistic regression estimation

For LR, training model to estimate the Beta coefficient of the all the independent variables, and use it as the weight of each evaluation index. The results of LR analysis are shown in Table 5. “Wald” represents the Wald chi-square value, which can be used to test the significance level of each variable. “Sig.” reflects the significance probability. In the study, the Sig values of DEM (P = 4.084E−05), Slope (P = 0.008), Rainfall (P = 1.056E−05), LULC (P = 8.222E−07) and Soil (P = 0.006) were less than 0.05, which indicated that these five predictors were statistically significant in the LR^85^. A positive value of Beta indicates that the variable is proportional to the probability of flooding, and vice versa^86^.

**Table 5:** Parameter of model.

	Beta	S.E.	Wald	df	Sig.	Exp(B)
DEM elevation	− 0.003	0.001	16.832	1	4.084e−05	0.997
Distance to rivers	− 0.001	0.001	0.981	1	0.322	0.999
Plan curvature	− 0.001	0.007	0.010	1	0.919	0.999
Slope	0.058	0.022	7.025	1	0.008	1.060
Aspect	0.001	0.001	1.278	1	0.258	1.001
Curvature	− 0.209	0.184	1.278	1	0.258	0.812
Rainfall	− 0.007	0.002	19.407	1	1.056e−05	0.993
SPI	0.103	0.085	1.476	1	0.224	1.108
STI	0.022	0.044	0.242	1	0.623	1.022
LULC	0.048	0.010	24.305	1	8.222e−07	1.049
Soil	0.006	0.002	7.653	1	0.006	1.006
TWI	0.015	0.062	0.060	1	0.806	1.015
(Constant)	37.831	8.929	17.952	1	2.265e−05	2.691 e+16

On the strength of the regression of all factors in Table 5, and according to Eq. (5), after removing the insignificant factors, the LR Equation is represented by Eq. (9):

9 $$\begin{aligned} {\text{Z}} & = \left( { - 0.003 \times {\text{DEM}}\;{\text{Elevation}}} \right) + \left( {0.058 \times {\text{Slope}}} \right) + \left( { - 0.007 \times {\text{Rainfall}}} \right) \hfill \\ & \quad + \left( {0.048 \times {\text{LULC}}} \right) + \left( {0.006 \times {\text{Soil}}} \right) + 37.831 \hfill \\ \end{aligned}$$

And then, substitute the z-value calculated above into Eq. (10) to calculate P:

10 $${\text{P}} = \frac{1}{{1 + {\text{e}}^{{ - {\text{z}}}} }} = \frac{1}{{1 + {\text{e}}^{{\left[ {\left( { - 0.003 \times {\text{DEM}}\;{\text{Elevation}}} \right) + \left( {0.058 \times {\text{Slope}}} \right) + \left( { - 0.007 \times {\text{Rainfall}}} \right) + \left( {0.048 \times {\text{LULC}}} \right) + \left( {0.006 \times {\text{Soil}}} \right) + 37.831} \right]}} }}$$

That is, the occurrence of flood is mainly explained by DEM, Slope, Rainfall, LULC and Soil.

### Interaction analysis

The true effect of one factor (the single effect) changes as the level of another factor changes. An interaction occurs when two or more exposure factors are present at the same time and the effect is not equal to the combined effect of their individual effects. The analysis of the interaction between the factors will help us to further understand the mechanism of their action on flood and their additive effect.

Interactions can be divided into additive interactions and multiplicative interactions There are two events, A and B, covering four categories: A−, A+, B− and B+.

In the additive interaction, If the relationship between A and B satisfies Eq. (11)

11 $${R}_{A+B+}-{R}_{A-B-}=\left({R}_{A+B-}-{R}_{A-B-}\right)+({R}_{A-B+}-{R}_{A-B-})$$

It indicates that there is no additive interaction between the two factors, where R is absolute risk (the same as below). If the relationship between A and B satisfies Eq. (12)

12 $${R}_{A+B+}-{R}_{A-B-}>\left({R}_{A+B-}-{R}_{A-B-}\right)+({R}_{A-B+}-{R}_{A-B-})$$

It indicates that there is a positive additive interaction between the two factors and vice versa.

In the multiplication interaction, If the relationship between A and B satisfies Eq. (13)

13 $${R}_{A+B+}/{R}_{A-B-}=\left({R}_{A+B-}/{R}_{A-B-}\right)\times ({R}_{A-B+}/{R}_{A-B-})$$

It shows that there is no multiplicative interaction between the two factors. If the relationship between A and B satisfies Eq. (14)

14 $${R}_{A+B+}/{R}_{A-B-}>\left({R}_{A+B-}/{R}_{A-B-}\right)\times ({R}_{A-B+}/{R}_{A-B-})$$

It indicates that the two factors have positive multiplication and interaction, and vice versa.

The index (RERI) evaluating the relative excess risk due to interaction is calculated with the Eq. (15) :

15 $$\begin{aligned} RERI & ={(R}_{A+B+}/{R}_{A-B-}-{R}_{A-B-}/{R}_{A-B-})-{(R}_{A+B-}/{R}_{A-B-}-{R}_{A-B-}/{R}_{A-B-}) \\ & \quad -{(R}_{A-B+}/{R}_{A-B-}-{R}_{A-B-}/{R}_{A-B-})=({RR}_{A+B+}-1)-({RR}_{A+B-}-1) \\ & \quad -({RR}_{A-B+}-1)={RR}_{A+B+}-{RR}_{A+B-}-{RR}_{A-B+}+1\end{aligned}$$

The attributable proportion due to interaction (AP) is calculated using Eq. (16):

16 $$AP=\frac{RERI}{{RR}_{A+B+}}$$

the synergy index S calculated using Eq. (17)

17 $$S=\frac{{RR}_{A+B+}-1}{{(RR}_{A+B-}-1)+{(RR}_{A-B+}-1)}$$

When there is no additive interaction between the two factors, the confidence interval of RERI and AP should contain 0, and the confidence interval of S should contain 1. In the LR used in this study, the condition satisfies Eq. (18)

18 $$logit\left(p\right)=\mathrm{ln}\left(\frac{p}{1-p}\right)=\mathrm{ln}\left(odds\right)={\beta }_{0}+{\beta }_{1}A+{\beta }_{2}B+{\beta }_{3}AB$$

The separate effect of A is expressed by Eq. (19) and (20):

19 $$\mathrm{ln}\left({odds}_{A+B-}\right)-\mathrm{ln}\left({odds}_{A-B-}\right)=\mathrm{ln}\left(\frac{{odds}_{A+B-}}{{odds}_{A-B-}}\right)=\mathrm{ln}\left({OR}_{A+B-}\right)={\beta }_{0}+{\beta }_{1}-{\beta }_{0}={\beta }_{1}$$

20 $${OR}_{A+B-}=\mathrm{exp}({\beta }_{1})$$

The separate effect of B is expressed by Eqs. (21) and (22):

21 $$\mathrm{ln}\left({odds}_{A-B+}\right)-\mathrm{ln}\left({odds}_{A-B-}\right)=\mathrm{ln}\left(\frac{{odds}_{A-B+}}{{odds}_{A-B-}}\right)=\mathrm{ln}\left({OR}_{A-B+}\right)={\beta }_{0}+{\beta }_{2}-{\beta }_{0}={\beta }_{2}$$

22 $${OR}_{A-B+}=\mathrm{exp}({\beta }_{2})$$

The combined effect of A and B is expressed by Eqs. (23) and (24):

23 $$\mathrm{ln}\left({odds}_{A+B+}\right)-\mathrm{ln}\left({odds}_{A-B-}\right)=\mathrm{ln}\left(\frac{{odds}_{A+B+}}{{odds}_{A-B-}}\right)=\mathrm{ln}\left({OR}_{A+B+}\right)={\beta }_{0}+{\beta }_{1}+{\beta }_{2}+{\beta }_{3}-{\beta }_{0} ={\beta }_{1}+{\beta }_{2}+{\beta }_{3}$$

24 $${OR}_{A+B+}=\mathrm{exp}({\beta }_{1}+{\beta }_{2}+{\beta }_{3})$$

The evaluation multiplication interaction is calculated using Eqs. (25) and (26):

25 $${RR}_{A+B+}/({RR}_{A+B-}\times {RR}_{A-B+})=\mathrm{exp}({\beta }_{1}+{\beta }_{2}+{\beta }_{3})/(\mathrm{exp}({\beta }_{1})\times \mathrm{exp}({\beta }_{2}))=\mathrm{exp}({\beta }_{3})$$

26 $$\left\{\begin{array}{l}\mathrm{exp}\left({\beta }_{1}+{\beta }_{2}+{\beta }_{3}\right)=\mathrm{exp}\left({\beta }_{1}\right)\times \mathrm{exp}\left({\beta }_{2}\right),{\beta }_{3}=0, \;\; \mathrm{ No \; multiplication \; interaction}\\ \mathrm{exp}\left({\beta }_{1}+{\beta }_{2}+{\beta }_{3}\right)>\mathrm{exp}\left({\beta }_{1}\right)\times \mathrm{exp}\left({\beta }_{2}\right),{\beta }_{3}>0, \;\; \mathrm{Positive \;multiplication\; interaction} \\ \mathrm{exp}\left({\beta }_{1}+{\beta }_{2}+{\beta }_{3}\right)<\mathrm{exp}\left({\beta }_{1}\right)\times \mathrm{exp}\left({\beta }_{2}\right),{\beta }_{3}<0, \;\; \mathrm{Negative \;multiplication \; interaction}\end{array}\right.$$

The evaluation additive interaction is calculated using Eqs. (27), (28) and (29):

27 $$RERI={RR}_{A+B+}-{RR}_{A+B-}-{RR}_{A-B+}+1=\mathrm{exp}\left({\beta }_{1}+{\beta }_{2}+{\beta }_{3}\right)-\mathrm{exp}\left({\beta }_{1}\right)-\mathrm{exp}\left({\beta }_{2}\right)+1$$

28 $$AP=\frac{RERI}{{RR}_{A+B+}}=\frac{\mathrm{exp}\left({\beta }_{1}+{\beta }_{2}+{\beta }_{3}\right)-\mathrm{exp}\left({\beta }_{1}\right)-\mathrm{exp}\left({\beta }_{2}\right)+1}{\mathrm{exp}\left({\beta }_{1}+{\beta }_{2}+{\beta }_{3}\right)}$$

29 $$S=\frac{{RR}_{A+B+}-1}{\left({RR}_{A+B-}-1\right)-\left({RR}_{A-B+}-1\right)}=\frac{\mathrm{exp}\left({\beta }_{1}+{\beta }_{2}+{\beta }_{3}\right)-1}{[\mathrm{exp}\left({\beta }_{1}\right)-1]+[\mathrm{exp}\left({\beta }_{2}\right)-1]}$$

When at least one of the two factors is a protective factor, the low-risk category is generally used as a reference (or the high-risk category is used as exposure).

In the construction of LR model, five factors are selected according to their significance, namely, DEM, Slope, Rainfall, LULC and Soil. In order to further explore the interaction between these factors, the above factors are analyzed by additive interaction and multiplication interaction (Table 6). Since only two categories can be used in the analysis of additive interaction, DEM, Slope and Rainfall are divided into two categories of high and low rainfall according to median and average, in order to further strengthen the influence of urbanization and human disturbance on flood sensitivity, The LULC is combined into urban building land (recorded as 1 in the calculation) and non-urban land (recorded as 2), and the soil type is integrated into non-human soil (recorded as 1) and man-made soil (recorded as 2).

**Table 6:** Analysis of interaction results.

	Individual effect	Common effect	Additive interaction	Multiplicative interaction
DEM	0.475			
Slope	0.809			
Rainfall	0.512			
Soil	26.27			
LULC	0.234			
DEM*slope		0.537		Positive multiplicative interaction
DEM*rainfall		0.234		Negative multiplicative interaction
Slpoe*rainfall		0.264		Negative multiplicative interaction
Soil*LULC		0.01		Negative multiplicative interaction

The results show that for product interaction, the individual effects of DEM, Slope and Rainfall are 0.475, 0.809 and 0.512 respectively. Slope has a greater influence on flood sensitivity, followed by Rainfall and DEM. When man-made soil and non-man-made soil are considered comprehensively, the possibility of flood is much higher than that of urban land and non-urban land. For the interaction of various factors, the multiplicative interaction between DEM and Slope promotes the occurrence of flood, while the interaction between DEM and Rainfall, Slope and Rainfall, Soil and LULC is negative. For additive interactions, RERI, AP and S are calculated, and the results show that there is no additive interaction.

### Validation of the susceptibility assessment results

**Figure 4:**
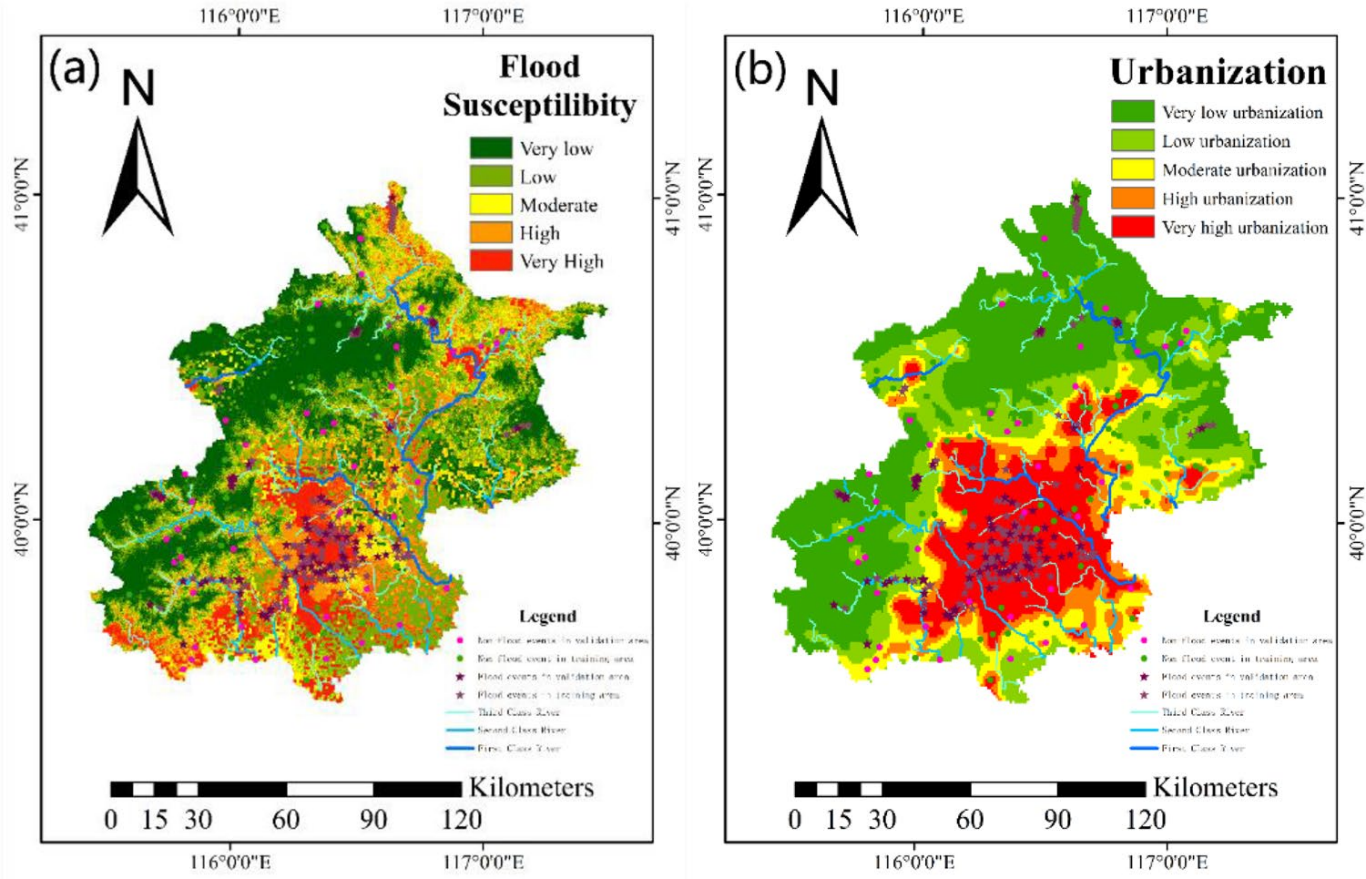
(**a**) Flood sensitivity map; (**b**) urbanization degree map (according to night light dataset) (we plotted the map using arcgis10.7. http://www.esri.com/sofware/arcgis).

**Figure 5:**
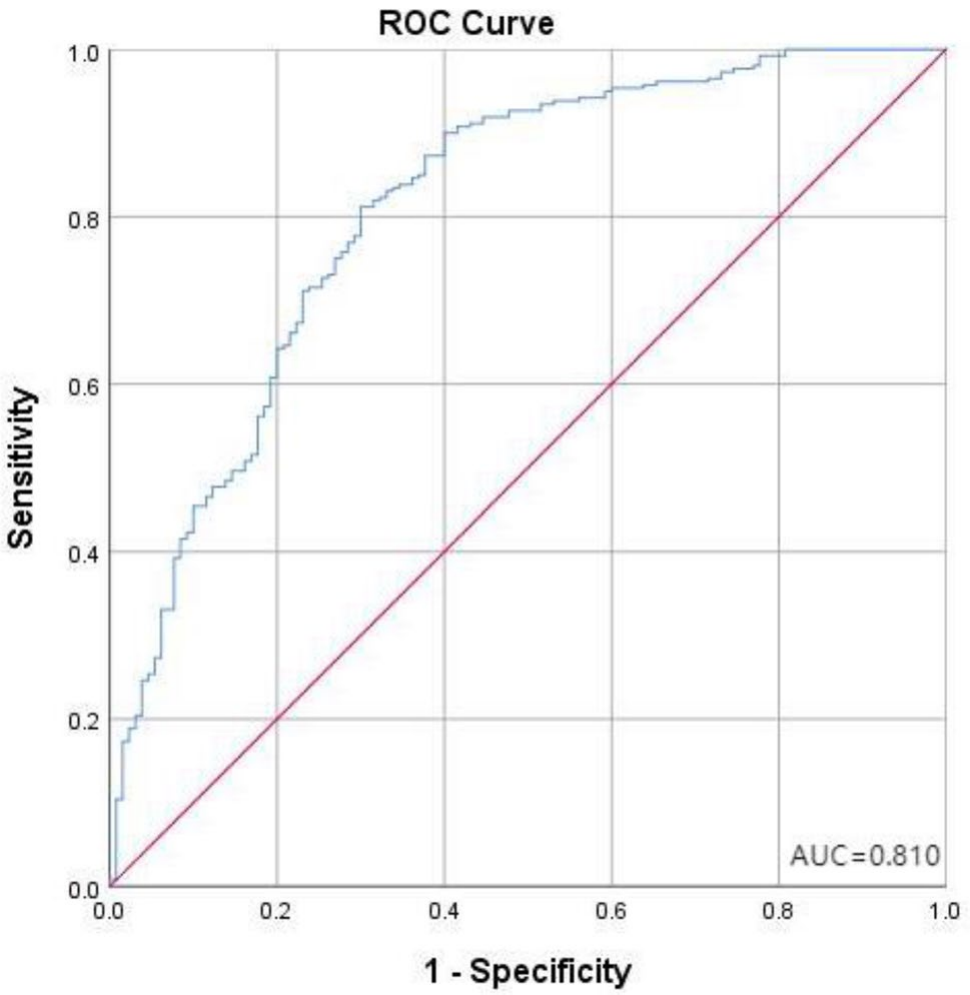
AUC value of the models for calibration and ROC curve.

During the flood sensitivity analysis to determine the future can be affected by the flood region is very important. In the study, LR were used to map flood sensitivity in a GIS environment (Fig. 4a)^87^. In order to verify the performance of the method, receiver operating characteristic (ROC) was used. The ROC model was developed based on graphs of true positive rates (sensitivity) versus false positive rates (1-specificity) with different cutoff points. The ROC curve was plotted by presenting the 1-specificity on the X-axis relative to the sensitivity on the Y-axis^24,88^. Where, sensitivity represents the total of pixels accurately divided into flood pixels, and 1-specificity represents several non-flood pixels. The AUC is measured by the area enclosed by the ROC curve (or broken line) and the horizontal axis, which was used as the evaluation criterion of the model^89^. The AUC can be calculated by Eq. (30):

30 $${\text{AUC}} = \frac{{\sum {{\text{TP}} + \sum {{\text{PN}}} } }}{{{\text{P}} + {\text{N}}}}$$

where, P and N represent the number of floods and non-floods, respectively. TP (true positive) and TN (true negative) indicate the number of pixels correctly classified. We consider AUC varying between 0.5 and 0.6 is a bad model. A range between 0.6 and 0.7 also indicates poor model performance, a range between 0.7 and 0.8 indicates medium model performance, and a value greater than 0.8 indicates very good model performance^90^. In this study, P = 260, T = 130, after learning and training of the model, the TP = 76, TN = 236, that is, 236 of the 260 actual flood points were predicted correctly, 24 were predicted incorrectly, and the prediction accuracy rate reached 90.8%. Of the 130 non-flood points, 76 were predicted correctly, and 54 were predicted incorrectly (Table 7). The comprehensive prediction accuracy of the model is 81%, which is represented by the lower surface surrounded by the product blue polyline in the ROC curve (Fig. 5). Based on this, we believe that the model has good prediction ability, but the prediction of non-flood points is still insufficient. The model overestimates the possibility of flood occurrence, which will lead to the government making unnecessary financial investment in flood control measures, but overestimating flood sensitivity has a higher guarantee for people's safety.

**Table 7:** Parameter of model.

		Predicted flood		
		0	1	Percentage correction (%)
Actual flood	0	76	54	58.5
	1	24	236	90.8
Total percentage				81.0

### Relationship between floods and susceptible areas

We classified flood sensitivity maps into five categories based on the natural breaks: very low (0–0.13), low (0.13–0.30), medium (0.30–0.48), high (0.48–0.65) and very high (0.65–1). Accounting for 29.49%, 27.70%, 15.36%, 14.38% and 13.06% of the study area, respectively. The very low, low and medium sensitive areas accounted for 72.56% of the total area, including 30.74% of the flood disaster points in the area of this study, and 27.44% of the areas were high and extremely high to flood disaster, including 70.43% of the flood disaster points (Table 8), indicating that the flood in these areas was densely distributed and highly sensitive. The model has a good degree of fitting and is consistent with the actual situation.

**Table 8:** Relationship between number of floods and level of susceptible areas.

	Susceptibility level	Number of floods	Flood ratio (%)	Partition area (km^2^)	Partition ratio (%)	Flood disaster density (1000 km^2^)
Flood susceptibility	Very low	19	7.39	4839.38	29.49	3.93
	Low	21	8.17	4546.03	27.70	4.62
	Moderate	39	15.18	2521.27	15.36	15.47
	High	72	28.02	2359.45	14.38	30.52
	Very high	109	42.41	2143.87	13.06	50.84
Urbanization	Very low	49	18.85	6589.49	40.16	7.44
	Low	28	10.77	3104.88	18.92	9.02
	Moderate	15	5.77	1978.16	12.05	7.58
	High	3	1.15	1422.36	8.67	2.11
	Very high	165	63.46	3315.11	20.20	49.77

The flood sensitivity map showed that the flood risk was highest in build-up areas of the study area. In addition, the northeast, north and southwest of Beijing are also at high risk of flooding. The lowest risk in the west. Through the comparison and analysis with the above 12 flood impact factors (Fig. 3), although there is relatively abundant precipitation in the west, but the region is mostly mountainous, with high altitude, which is not prone to flood disasters, and the aspect of the region is mainly northwest. Even if a flash flood does occur, it will travel northwest, away from the main urban area. Elevation and aspect are inversely correlated with the contribution of rainfall to the sensitivity, which may inhibit the positive effect of precipitation on the sensitivity to flood.

## Discussion

Accurate estimates of flood sensitivity are key to keeping people safe and developing effective mitigation measures. However, many factors control the development of flooding, it can never be fully predicted. Therefore, we suggest that flood sensitivity assessment should be carried out for each city, and preparations should be made in advance in flood prone areas to deal with possible future emergencies^91^. Similarly, select the appropriate evaluation indexes, the perfect forecasting model, improve the accuracy of the susceptibility evaluation result is very important. Through the literature, a growing number of single machine learning algorithms, integrated hybrid algorithm used for flood sensitivity modeling, this study still use the traditional LR and combined with the GIS and RS, and obtained better performance verification, simple and efficient, which can help managers make decisions on flood control and megacity development. Table 9 shows the flood impact factors and variable categories. Among the 12 factors in total, five factors with significance less than 0.05 were selected as the model parameters by Pearson correlation coefficient test, multicollinearity test and other steps: DEM, Slope, Rainfall, LULC and Soil. This is not the same as the previous research results, because we integrate flash floods and waterlogging into a whole for research. Both them are natural disasters faced by super cities, so the flood inventory drawn in this study covers both. The result of analysis is the flood sensitivity of super cities under the combined effects of flash flood and waterlogging. The flood sensitivity map and Table 9 can clearly reflect the occurrence of floods in different values/categories of each factor, and the flood hazard density can be used to assess the flood susceptibility.

**Table 9:** Flood conditioning factors and variable classes.

Factor	Classification	Number of floods	Flood ratio (%)	Partition area (km^2^)	Partition ratio (%)	Flood disaster density (1000 km^2^)
DEM^a^ (m)	4–180	118	64.48	7358.43	44.84	16.04
	180–438	32	17.49	2601.04	15.85	12.30
	438–714	24	13.11	3461.97	21.10	6.93
	714–1068	8	4.37	2321.99	14.15	3.45
	1068–2298	1	0.55	666.57	4.06	1.50
Slope^a^ (degree)	0–6.22	91	49.73	7059.37	43.02	12.89
	6.22–15.11	46	25.14	3074.03	18.73	14.96
	12.11–24.59	20	10.93	2787.24	16.98	7.18
	24.69–34.96	17	9.29	2390.47	14.57	7.11
	34.96–75.56	9	4.92	1098.89	6.70	8.19
LULC^a^	Cultivated land	10	5.46	3625.63	22.09	2.76
	Forest land	53	28.96	7516.65	45.81	7.05
	Grassland	9	4.92	1287.47	7.85	6.99
	Water area	3	1.64	439.54	2.68	6.83
	Town	108	59.02	3524.72	21.48	30.64
	Unused	0	0.00	15.98	0.10	0.00
Soil^a^	Brown soil	3	1.64	1400.73	8.54	2.14
	Cinnamon	96	52.46	9701.69	59.12	9.90
	Newly accumulated soil	0	0.00	14.00	0.09	0.00
	Aeolian soil	0	0.00	14.97	0.09	0.00
	Rocky soil	0	0.00	63.39	0.39	0.00
	Coarse bone soil	6	3.28	1026.68	6.26	5.84
	Black soil	0	0.00	49.87	0.30	0.00
	Moutain meadow soil	0	0.00	12.08	0.07	0.00
	Tidal soil	42	22.95	3739.37	22.79	11.23
	Paddy soil	3	1.64	53.28	0.32	56.30
	Town	31	16.94	184.72	1.13	167.82
	Water	2	1.09	149.24	0.91	13.40
Rainfall^a^ (ml)	534.52–563.61	16	8.74	1481.51	9.03	10.80
	563.61–574.65	51	27.87	4438.95	27.05	11.49
	574.65–583.86	114	62.30	7645.86	46.59	14.91
	583.86–594.54	2	1.09	2058.94	12.55	0.97
	594.54–628.42	0	0.00	784.73	4.78	0.00
Distance to rivers (M)	0–5187.96	36	19.67	4667.34	28.44	7.71
	5187.96–10,894.73	53	28.96	4533.97	27.63	11.69
	10,894.73–17,120.29	46	25.14	3778.35	23.02	12.17
	17,120.29–24,729.31	48	26.23	2421.99	14.76	19.82
	24,729.31–44,097.72	0	0.00	1008.35	6.14	0.00

^a^State the final parameter in the LR model.

For the five factors with significance less than 0.05: (1) the flood disaster density at the lower elevation (4–180 m) is high, which is mainly concentrated in the build-up area and low-lying valley. In a rainstorm event, the water flow at the upper level quickly gathers and rushes to the lower level, and a large number of flowing water from the upstream leads to the rapid rise of the water level and velocity at the downstream, which is the generation of flash flood. In addition, cement and asphalt with poor permeability serve as the underlying surface, which makes the water infiltration speed slow, and the upstream water and rainfall accumulate in the low-lying areas, which is the occurrence of waterlogging. (2) More floods occur when the slope is gentle (0°–6.22°, 6.22°–15.11°), which is mainly because waterlogging occurs in flat areas with depressions. When the slope is steep, floods occur in each value range, and the flood disaster density is basically the same. (3) Rainfall is basically proportional to flood disaster density, but less floods occur in areas with the most rainfall (583.86–594.54 ml, 594.54–628.42 ml). This may be because we are not comprehensive enough in drawing the flood inventory map or under the comprehensive influence of other factors. These areas are less prone to flooding even with high rainfall, or are surrounded by multiple mountain ranges that impede atmospheric circulation. (4) For LULC, the most prominent is that there are more flood disasters in the main urban area, and the flood disaster density is 30.64, followed by woodland, grassland and water area, and the flood disaster density is 7.05, 6.99 and 6.83, respectively. (5) The influence of soil on flood also reflected that the flood disaster density was high in the built-up area, and the underlying surface was mainly cement and asphalt, followed by paddy soil. Paddy soil is one of the most important tillage soils in China. The above findings also remind us that we should always pay great attention to the main urban areas. Discussion on the interaction of various factors, For the same DEM, a larger Slope reduces the likelihood of flooding, and for the same Slope, a higher DEM reduces the likelihood of flooding. This is because waterlogging occurs more in the study area, the urban terrain is flat, the elevation is low, the slope is small, and the drainage capacity is poor, which is easy to waterlog disaster. For the same DEM or Slope, more abundant rainfall reduces the possibility of flood, which is different from our previous cognition. However, according to the analysis in Fig. 3l, the precipitation distribution in the study area has obvious spatial limits, and the areas with heavy rainfall are all distributed in mountainous areas. This may be due to the heat island effect caused by supercities and the extensive vegetation in mountainous areas, which increases local water vapor content and thus local rainfall. Therefore, less precipitation brings more flooding disasters in low-lying urban areas than in mountainous areas. In the range of non-human soil, the flood disaster of non-urban land is low, the flood disaster of urban land is high, and vice versa.

According to the common sense, the density and use of lighting facilities can reflect the prosperity of the area. Therefore, light intensity and light density reflected by night light data can reflect the degree of urbanization and the distribution of population density. By using the natural discontinuous point method, we divided the obtained and processed night light data into five categories, and counted the flood disaster densities of different degrees of urbanization in Fig. 4b and Table 6. By comparing the images and statistical tables, we can find that the flood disaster density in the very high area is the highest in both the urbanization degree and the flood sensitivity degree, and far exceeds other levels. In the Logistic regional estimation, we found a relatively abnormal result. The previous research showed that "the closer to the river, the greater the flood sensitivity". In this study, although "distance to rivers" is not a significant influencing factor, it is negatively related to flood sensitivity, which seems to be contrary to common sense. We believe that the reason for this result may be that there are more waterlogging disasters within the time series studied. There are fewer rivers in the main urban area, and they are usually subject to the flood control treatment of heightening dikes, so it is not easy to flood due to the rise of river water level. Therefore, the results of this study appear.

The more urbanized the region, the greater the risk of flood. The development of super cities shows a trend of radiating and expanding from the city center to the outside, and finally realizing the overall urbanization. The flood sensitivity of each region also increases. Therefore, we should always pay attention to the regions where floods may occur in the future.

Compared with other machine learning algorithms, LR model has the following disadvantages: (1) When the feature space is large, the performance of logistic regression is not very good. (2) Can not handle a large number of multi-class features or variables well. (3) For nonlinear features, transformation is required. (4) Compared with the more complex model, the training effect is poor. Even so, due to the fast training speed of LR model and the good interpretability of model, users do not have to worry about whether the features of data are related as in naive Bayes. Compared with decision tree and SVM, it can also get a better probability interpretation, and can easily use new data to update the model and a series of advantages. In addition, from the perspective of practicability, this study chooses this model for prediction analysis, and the results also show that it has satisfactory prediction ability.

In my opinion, there are still the following deficiencies in our study which deserve further improvement: (1) We used free low-resolution datasets in the acquisition of LULC, Soil and Rainfall predictors. Although they were resampled, they still had an impact on the accuracy of prediction results. In the following study, higher precision datasets can be used. (2) Due to insufficient data acquisition, the 260 flood points used in this study could not cover all the floods in the 10-year time series in a large study area, which may have an impact on the significance of the predictors. (3) Future studies should be conducted with higher precision in areas with high susceptibility to provide more convincing opinions to policy makers.

## Conclusion

Identification of flood-prone areas is indispensable for watershed and land administration, especially for the protection of people's property and life safety. After identifying flood-prone areas, both managers and people living in high-risk areas should be vigilant during the rainy season and pay attention to possible flood disasters^92^. In the study, RS were used to identify areas susceptible to flooding in GIS environment by LR model, and a flood sensitivity map of Beijing was created. The 260 flood points used in this study included 130 flash flood points and 160 waterlogging points, and flash flood and waterlogging were taken as a whole for sensitivity assessment. Flood points are randomly subdivided into two parts, one for training points (70%) and one for model building and testing (30%)^92,93^. Pearson test, multicollinearity test and other steps were then performed to ensure that the factors plugged into the model were valid. Finally, AUC was used to evaluate the performance of the model. We use the LR model of one big advantage of this method is easy to understand, don't need any specific software or complicated programs. The main conclusions of our research summarized below:

1. DEM, Slope, Rainfall, LULC and Soil were significant at 95% confidence interval, which greatly influenced the occurrence of flood. Under the separate action of each factor, Slope has a greater influence on flood sensitivity, followed by Rainfall and DEM. There is no additive interaction among the above forecasting factors that have significant influence on flood; the multiplicative interaction between DEM and Slope promotes the occurrence of flood, while there is negative interaction between DEM and Rainfall,Slope and Rainfall, Soil and LULC.
2. The AUC value is greater than 0.8, and the model is considered to have good predictability. The reliability of LR model is proved again through this research.
3. The proportion of high risk and extremely high risk areas was 27.44%, including 70.43% of the flood events, which were mainly distributed in urban areas with a high degree of urbanization, indicating that flood hazards are densely distributed in these areas and are highly sensitive.
4. No matter in terms of urbanization degree or flood sensitivity degree, the flood disaster density in the very high region is the highest, which is 49.77 and 50.84 respectively, and far exceeds other grades. This also indicates that super cities will face higher and higher flood risks in the process of radiating outward from the main urban area as the center, and disaster prevention and control should be done well in the urban construction.

There will be more and more super cities around the world. The conclusion of this study wants to show that in the process of super cities reconstruction and construction, we should keep a high alert to potential flood disasters, pay attention to disaster prevention and control, and strengthen the deployment of drainage, waterlogging and flood fighting, so as to escort urban development and people's safety.

## Data Availability

The data that support the findings of this study are openly available in [Resource and Environmental Science and Data Center] at [https://www.resdc.cn/Default.aspx].
